# HLA DQ2/DQ8 haplotypes and anti-transglutaminase antibodies as celiac disease markers in a pediatric population with type 1 diabetes mellitus

**DOI:** 10.20945/2359-3997000000457

**Published:** 2022-04-19

**Authors:** Diana Rita Oliveira, Joana Freitas Rebelo, Cristiana Maximiano, Maria Miguel Gomes, Vânia Martins, Carla Meireles, Henedina Antunes, Sofia Martins

**Affiliations:** 1 Hospital de Braga Braga Portugal Serviço de Pediatria, Hospital de Braga, Braga, Portugal; 2 Faculdade de Medicina da Universidade do Minho Braga Portugal Faculdade de Medicina da Universidade do Minho, Braga, Portugal; 3 Hospital de Braga Departamento de Pediatria Unidade de Endocrinologia e Diabetologia Pediátrica Braga Portugal Unidade de Endocrinologia e Diabetologia Pediátrica, Departamento de Pediatria, Hospital de Braga, Braga, Portugal; 4 Centro Hospitalar Trás-os-Montes e Alto Douro Serviço de Pediatria Vila Real Portugal Serviço de Pediatria, Centro Hospitalar Trás-os-Montes e Alto Douro, Vila Real, Portugal; 5 Hospital Senhora da Oliveira-Guimarães Guimarães Portugal Serviço de Pediatria, Hospital Senhora da Oliveira-Guimarães, Guimarães, Portugal; 6 Unidade de Gastroenterologia Braga Portugal Unidade de Gastroenterologia, Hepatologia e Nutrição, Serviço de Pediatria e Centro Académico Clínico Hospital de Braga, Braga, Portugal; 7 Faculdade de Medicina da Universidade do Minho Instituto de Investigação em Ciências da Vida e da Saúde Braga Portugal Instituto de Investigação em Ciências da Vida e da Saúde (ICVS), ICVS/3B's-PT Laboratório Associado do Governo e Faculdade de Medicina da Universidade do Minho, Braga, Portugal

**Keywords:** Type 1 diabetes, celiac disease, HLA, transglutaminase, pediatric age

## Abstract

**Objectives:**

Evaluate the celiac disease (CD) markers, within the scope of its screening, in a pediatric population with diagnosis of type 1 diabetes (T1D) at Hospital de Braga (HB) and determine the prevalence of CD in the sample. Reflect on CD screening algorithm applied in this pediatric population.

**Subjects and methods:**

Retrospective observational study with 94 patients diagnosed with T1D at age 10 years or younger, followed up at the HB Outpatient Diabetology Consultation, including those referred from other hospitals. Record of clinical information, IgA anti-transglutaminase and anti-endomysium and HLA DQ2/DQ8 haplotypes.

**Results:**

We obtained positive serological test for CD in 4 patients. This test had 100% sensitivity and specificity. The prevalence of CD was 4.3% (n = 4). Positive HLA screening in 84.6% of patients, with both sensitivity and negative predictive value of 100% and specificity of 16.67%. Diagnosis of CD was made on average 3.40 ± 3.32 years after the diagnosis of TD1. All cases of CD registered non-gastrointestinal manifestations, none had gastrointestinal symptoms.

**Conclusion:**

This study proved that there is a higher prevalence of CD in pediatric population with TD1, when compared to general population, and clarified the importance of CD screening. Furthermore, it was observed that serological screening for CD antibodies is an excellent screening test and HLA typing, although not the most suitable first line test, can be useful in excluding the possibility of patients with T1D developing CD.

## INTRODUCTION

Type 1 diabetes mellitus (T1D) corresponds to 90% of all diagnoses of diabetes in pediatric age and is defined as a chronic autoimmune disease due to an immune-mediated destruction of insulin-producing β-cells of the pancreas ( 1 ). In accordance with its etiology, children with T1D are at increased risk of developing other autoimmune diseases (AID) when compared to general population, among which autoimmune thyroiditis (AIT) stands out as the most common, followed by celiac disease (CD) ( 2 – 7 ).

CD is a chronic AID characterized by serological and histological changes in the small intestine of individuals with genetic predisposition that, when exposed to some environmental agents, develop an immune response triggered by the ingestion of gluten ( 8 – 10 ).

The prevalence of CD in children and adolescents with T1D varies between 1.6%-10%, compared to 1% recorded in general population ( 2 , 4 , 11 – 14 ). A study in a Portuguese pediatric population showed a prevalence of 1:134 ( 15 ). The diagnosis of CD can precede the diagnosis of T1D, however in 90% of cases T1D is diagnosed first. Often, when a patient has both diseases, the diagnosis of CD occurs up to 5 years after the diagnosis of T1D ( 16 – 18 ). The coexistence of these two diseases can be justified by shared risk factors, namely genetic, environmental, and pathophysiological mechanisms ( 5 , 11 , 19 – 21 ).

As for the genetics, the human leukocyte antigen (HLA) system DR-DQ seems to be responsible for 40%-50% of the genetic risk of developing T1D and 53% in the case of CD ( 22 ). In T1D HLA DR3-DQ2/DR4-DQ8 represents the greatest risk factor, while in CD is HLA DR3-DQ2/DR3-DQ2 ( 23 – 25 ). These genotypes, especially HLA DR3-DQ2/DR4-DQ8, are also responsible for determining a higher risk for coexistence of these diseases in the same person ( 19 , 22 , 25 – 27 ). These genes encode protein complexes present on the surface of antigen-presenting cells that bind to β-cell islet autoantigens and specific gluten fragments, respectively, by presenting them to reactive CD4 + T-cells. Thus, in an inflammatory environment, autoimmune destruction of β cells in the pancreas and/or intestinal enterocyte occurs ( 20 , 26 ). Genetic predisposition proves to be a necessary, but not sufficient condition to develop CD, despite 25%-35% of the population is positive for these alleles only 3% will develop the disease ( 7 , 8 ). Currently, we believe that environmental factors play an important role in the development of CD, namely viral infections, dysregulation of the intestinal microbiome and diet containing gluten ( 9 , 10 )

CD clinically presents with gastrointestinal (GI) and non-GI symptoms. Classical/intestinal symptoms include constipation, chronic diarrhea, steatorrhea, abdominal pain and distension, nausea and vomiting ( 28 ). Non-GI manifestations include signs and symptoms such as weight loss, chronic fatigue, neuropathy, iron deficiency anemia, osteopenia/osteoporosis, arthralgia/arthritis, pubertal delay, and amenorrhea ( 8 , 28 ). In the case of children and adolescents with T1D, the clinical presentation of CD is characterized by moderate non-GI symptoms, or even asymptomatic.

Some studies have shown a higher prevalence of retinopathy and nephropathy in adults with T1D and CD and a 2.8 times higher mortality risk in T1D patients diagnosed with CD for at least 15 years ( 29 , 30 ). Thus, periodic screening for this disease in this population is essential ( 19 , 21 , 26 , 31 – 33 ).

According to the most recent guidelines from the American Diabetes Association (ADA) and the International Society for Pediatric and Adolescent Diabetes (ISPAD), screening for CD in children or adolescents with T1D should be done at the time of diagnosis of T1D and, subsequently, after 2 and 5 years ( 2 , 34 ). Recommendations for serological screening of CD have been changing in the past years, with the most recent ESPGHAN guidelines published in 2020. The anti-gliadin antibody, previously the serologic test of choice, is no longer recommended due to its lower sensitivity and specificity. Testing for endomysial antibodies (EmA) as initial screening for CD is currently not recommended, despite its high specificity, not only because the interpretation of the results is subjective, but also because the immunofluorescence technique is more expensive and time-consuming than measurement of transglutaminase (atTG). A study publish by the American Family Physician also states that EmA screening has a lower sensitivity than atTG, with a positive predictive value of 79% ( 35 ). The screening for CD is based on total immunoglobulin A (IgA), IgA anti-transglutaminase (anti-atTG) and/or anti-endomysium (Anti-EmA) antibodies, except for cases of IgA deficit, in which immunoglobulin G (IgG) antibody assays are used ( 2 ). If at any point during the screening the antibodies are positive, an upper digestive endoscopy (UDA) should be performed in which at least 5 biopsies are performed: 4 duodenal and 1 bulb ( 28 ). If the histological results meet the criteria for CD defined in the Modified Marsh classification, the diagnosis is confirmed, and these patients are excluded from this screening ( 2 , 28 , 36 ). If anti-tTG IgA value is at least 10 times greater than the upper normal limit, in the presence of positive anti-EMA IgA collected in a different sample, the diagnosis can be made without biopsy ( 2 , 28 ).

As for the HLA DR3-DQ2 and DR4-DQ8 haplotypes, there is still no consensus. The new guidelines from the European Society for Pediatric Gastroenterology Hepatology and Nutrition (ESPGHAN) state that HLA research is not necessary in patients with positive antibodies that meet CD criteria either by biopsy or by anti-tTG IgA titer and assumes that development of CD is unlikely if both alleles are absent. On the other hand, ISPAD doesn't recommend HLA typing, considering it impractical and not cost-effective as a first-line screening test for CD, though it rarely allows the exclusion of CD in patients with T1D ( 2 , 28 ).

This study aims to evaluate the CD markers, namely the antibodies and haplotypes associated, and the prevalence of this disease in a pediatric population with T1D followed up at Diabetology consultation in Hospital de Braga (HB) and referred from other hospitals and to reflect on the CD screening algorithm applied in this population.

## SUBJECTS AND METHODS

Retrospective, observational, and analytical study that focused on all children with T1D diagnosed up to 10 years of age, inclusive, followed up in a pediatric diabetology consultation in HB between January 2009 and January 2020. We selected 115 patients, 21 patients were excluded due to the absence of a serology information registration for CD antibodies at the time of the diagnosis of DM1, resulting in a total of 94 patients.

The patients included in the study population were divided into 2 groups, according to the diagnosis of CD: group 1 without CD and group 2 with CD. Diagnosis of CD was confirmed if positive serology with positive histological biopsy result. The serological result of CD antibodies, obtained at the diagnosis of T1D and after 2 and 5 years, was considered positive if at least one of the autoantibodies, anti-EmA and/or anti-tTG, IgA or IgG, was positive. Regarding the duodenal endoscopic biopsy, those who met the criteria described in types 2 and 3 of the Modified Marsh Classification were assumed as positive histological results ( 35 ). The result for HLA DR3-DQ2 and DR4-DQ8 was considered positive when at least one of the alleles was present.

Patients' sociodemographic and clinical data, as well as family history, were obtained by consulting the electronic clinical process. Family history and the patient's past history of AID, T1D and the associated AIDs were included: IAT, CD, inflammatory bowel disease (IBD), autoimmune hepatitis, autoimmune gastritis, primary adrenal insufficiency, rheumatoid arthritis (RA), systemic lupus erythematosus (SLE), psoriasis, scleroderma and vitiligo ( 2 ).

Considered GI symptoms were: intermittent or chronic diarrhea, constipation, abdominal pain, abdominal distension, nausea and recurrent vomiting and non-GI manifestations were: poor weight gain, pubertal delay, amenorrhea, chronic fatigue, irritability, neuropathy, arthralgias/arthritis, recurrent aphthous stomatitis, herpetiform dermatitis, nail changes, iron deficient anemia, repetitive bone fractures, decreased bone mineralization and abnormal liver biochemistry ( 28 ).

All patient's data were recorded in an anonymized database created for this purpose.

### Statistical analysis

Statistical analysis with the International Business Machine^®^ Statistical Package for the Social Sciences^®^ (IBM-SPSS) Statistics software. A significance level of 5% was established, statistical significance defined as p < 0,05.

Descriptive study of the two groups (group 1 and 2) with additional comparison between them was performed. Normality was tested for quantitative variables, which assumption underlies the use of parametric statistics, and this analysis was based on the values of asymmetry and kurtosis, the results of the Kolmogorov-Smirnov and Shapiro-Wilk tests, the histogram analysis and the QQ graph ( 36 ). For the quantitative variables that didn't have a normal distribution, parametric and nonparametric tests were performed, obtaining the same results so the results of the parametric tests were reported ( 37 ).

Qualitative variables were described in absolute value (n) and percentage (%), mean (M) and standard deviation (SD) were used as descriptive measures. Chi-square test (χ2) was used to compare qualitative variables of the two groups. However, the percentage of cells with an expected count below 5 was always greater than 20%, so Fisher's Exact test was reported. Phi (Φ) was used as an effect size measure.

Comparison of quantitative variables between groups using the *t* -test for independent samples (t), and the assumption of homogeneity of variances was assessed using the Levene test. As a measure of effect size, Cohen's d value (d) was calculated, considering the intervals 0.20 and 0.50; 0.50 and 0.80; and greater than 0.80 as weak, medium, and strong, respectively ( 38 ).

The evaluation of the screening tests' characteristics was based on the following formulas: sensitivity = number of individuals with CD with positive test/total number of individuals with CD; specificity = number of individuals without CD with negative test/total number of individuals without CD; positive predictive value (PPV) = probability of an individual evaluated and with a positive result actually having CD; negative predictive value (NPV) = probability of an individual evaluated and with a negative result not having CD.

## RESULTS

We selected 115 patients followed up in Pediatric Diabetology consultation in HB, diagnosed with DM1 before 10 years of age. According to the previously described criteria, 21 patients were excluded. Of the 94 patients included in the sample, 52.1% were female (n = 49), with a mean age of 5.96 ± 3.81 years. The mean age at diagnosis of DM1 was 5.94 ± 2.57 years, with a mean follow-up time of 4.57 ± 2.94 years. Past history of AID in 3.2% of cases (3 AIT) and family history in 14.9% (6 T1D; 3 SLE; 3 AIT; 2 CD; 1 Psoriasis and T1D). The characterization is shown in Table 1 .

**Table 1:** Sample description

		N = 94
Gender, n (%)		
	Female	49 (52,1%)
	Male	45 (47,9%)
Age (years)		
	Mean	5,96
	SD/Min-Max	3,811/1-18
Age at T1D diagnosis (years)		
	Mean	5,94
	SD/Min-Max	2,57/1,08-10,75
Follow-up of T1D (years)		
	Mean	4,57
	SD/Min-Max	2,94; 0,08 10,08
PH of AID n (%)		3 (3,2%)
	AIT	3 (100%)
FH of AID n (%)		14 (14,9%)
	T1D	6 (42,86%)
	SLE	3 (21,43%)
	CD	2 (14,29%)
	Psoríase e and T1D	1 (7,14%)
	AIT	2 (14,29%)
HLA screening, n (%)		
	Negative	8 (15,4%)
	Positive	44 (84,6%)
IgA deficiency, n (%)		1 (1,1%)
Antibodies IgA/IgG at diagnosis, n (%)		
	Negative	90 (95,7%)
	Positive	4 (4,3%)
Antibodies IgA/ IgG (2 years) n (%)		
	Not applicable	15 (16,7%)
	Negative	75 (83,3%)
Antibodies IgA/IgG (5 years) n (%)		
	Not applicable	28 (31,1%)
	Negative	62 (68,9%)
Biopsy histology n (%)		
	Number of biopsies	4 (4,3%)
	Positive	4 (100%)
CD diagnosis n (%)		
	Negative	90 (95,7%)
	Positive	4 (4,3%)

AID: autoimmune disease; AIT: autoimmune thyroiditis; CD: celiac disease; FH: family history; PH: past history; SLE: systemic lupus erythematosus.

As for HLA typing, 42 patients didn't present any record and, in the remaining, 84.6% had a positive result. At the diagnosis of T1D 4.3% (n = 4) of the patients presented positive serology for CD, with diagnostic confirmation by compatible duodenal biopsy according to the Modified Marsh classification ( 35 ). Thus, CD was diagnosed in 4 patients, with a prevalence of 4.3%. Among the cases with positive serology, one of them had an IgA deficit and IgG antibodies were assayed and came back positive.

### Comparison between the two groups of patients with DM1

Positive serology for CD antibodies was significantly higher in the group with CD compared to the group without CD (p = 0.001; Φ = 1.00). There were no statistically significant differences between the two groups for the remaining variables. There was a higher prevalence of females (52.2%) in the group without CD, while in the group with CD the ratio between genders was 1: 1 (p = 1,000; Φ = 0.85). In the group with CD, the average age at diagnosis of T1D (7.88 ± 2.23 years) was higher than in the group without CD (5.85 ± 2.56 years) (p = 1,000; d = 0.01). Only the group without CD had a personal history of AID (p = 1,000; Φ = −0.38). As for family history of AID, this was higher in the group of patients with CD compared to the group without CD (p = 0.104; Φ = 0.21). HLA positivity was more frequent in the group with CD (100% vs. 83.7%; p = 1,000; Φ = 0.11).

### Characterization of CD presentation in patients with T1D

The characterization of the presentation of CD in patients with T1D is shown in Table 3 . Average age at diagnosis of CD was 10.50 ± 2.65 years. The follow-up time until the diagnosis of CD was on average 3.40 ± 3.32 years after the diagnosis of T1D. HLA typing was positive in all cases with records obtained (n = 3; 2 positive HLA DQ2/ DQ8, 1 HLA DQ2), as well as positive serology for CD antibodies (n = 4; 1 anti-tTG IgA, 2 Anti-EmA and anti-tTG IgA, 1 anti-EmA and anti-tTG IgG). All cases with type 3 histology according to Modified Marsh classification (n = 4; 2 3B, 2 3C). As for clinical manifestations of CD, all presented exclusively non-GI symptoms (n = 4; 1 poor weight gain, 1 nail changes, 1 iron deficient anemia, 1 weight loss and hypoglycemia).

**Table 2:** Distribution of the variables under study for 2 separate groups considering the diagnosis of CD

		CD diagnosis				
		Without CD (n = 90)	With CD (n = 4)	Statistics	p	Effect size
Gender, n (%)				Fisher	1,000	Φ = 0,01
	Female	47 (52,2%)	2 (50,0%)			
	Male	43 (47,8%)	2 (50,0%)			
Age at T1D diagnosis (years)				t(92) = −1,550	1,000	d = 0,01
	Mean	5,85	7,88			
	SD/Min-Max	2,56/1,08-10.42)	2,23/5,67-10,75			
PH of AID, n (%)				Fisher	1,000	Φ = 0,38
	No	87 (96,7%)	4 (100%)			
	Yes	3 (3,3%)	0 (0%)			
FH of AID, n (%)				Fisher	0,104	Φ = 0,21
	No	78 (86,7%)	2 (50%)			
	Yes	12 (13,3%)	2 (50%)			
HLA screening, n (%)				Fisher	1,000	Φ = 0,11
	Negative	8 (16,3%)	0 (0%)			
	Positive	41 (83,7%)	3 (100)			
CD antibodies, n (%)				Fisher	0,001 *	Φ = 1,00
	Negative	90 (100%)	0 (0%)			
	Positive	0 (0%)	4 (100%)			

* P < 0.05;

Φ = Phi; Cohen's d - d.

**Table 3:** Characterization of CD presentation in patients with T1D

Age at diagnosis of CD (years)		
	Mean	**10,50**
	SD/Min-Max	**2,65/7,0-13,0**
Follow-up between T1D and CD diagnosis (years)		
	Mean	3,40
	SD/Min-Max	3,32/0,08-7,25
HLA screening, n (%)		
	Registered	3 (75%)
	HLA DQ2	1 (33,3%)
	HLA DQ2/DQ8	2 (66,7%)
Positive IgA antibodies (at diagnosis), n (%)		
	Anti-EmA IgA	0 (0%)
	Anti-tTG IgA	1 (33,3%)
	Anti-EmA e Anti-tTG IgA	2 (66,7%)
Positive IgG antibodies (at diagnosis), n (%)		
	Anti-EmA e Anti-tTG IgG	100%, n = 1
Biopsy histology n (%)		
	3B	2 (50%)
	3C	2 (50%)
GI symptoms n (%)		0 (0%)
Non-GI symptoms n (%)		4 (100%)
Poor weight gain		1 (25%)
Nail changes		1 (25%)
Iron deficient anemia		1 (25%)
Poor weight gain and hypoglycemia		1 (25%)

### Characterization of the tests applied for screening for CD

Data are described in Table 4 . Regarding serology for CD antibodies, all properties of this test showed a percentage of 100%. As for HLA screening, this test revealed a sensitivity and VPN of 100%, specificity of 16.67% and PPV of 9.09%.

**Table 4:** Properties of the tests included in the DC screening

	Serology	HLA screening
Sensitivity	100%	100%
Specificity	100%	16,6%
PPV	100%	9,09%
NPV	100%	100%

## DISCUSSION

The prevalence of CD in our study was 4.3%, consistent with that described in the literature, which demonstrates that the prevalence of CD in pediatric patients with T1D (1%-10%) is higher than in general population (1%) ( 2 , 13 , 16 ). Our findings are also in agreement with a study published by Yasin Sahin that also found a prevalence of 4,4% of CD in children with T1D ( 37 ). More recently, a cross-sectional study published in October 2020, that collected data from 57375 pediatric patients with T1D from the SWEET database (Better control in Pediatric and Adolescent diabetes): Working to crEate cEnTers of Reference), obtained similar data for DC prevalence (4.5%) ( 38 ).

In our study, the serology for CD was positive in 4.3%, similar to the results reported by the Birmingham Pediatric Hospital in a retrospective study in 2009, which included 555 children with T1D, and demonstrated a 3.9% positivity ( 39 ).

We also found that sensitivity and specificity of serology for CD antibodies was 100%. This data is supported by other studies that demonstrated a sensitivity and specificity of more than 90% ( 40 , 41 ). Thus, it was possible to infer that this test has properties that classify it as a good screening test.

Regarding the genetic component of this screening, 84.6% of the sample had a positive result. This test revealed a sensitivity of 100% and a specificity of 16.67%. This study supports the perspective of previously published articles that the utility of HLA typing as first line test in screening for CD in patients with T1D is limited, since both diseases share the same genetic predisposition ( 17 , 42 – 44 ). This topic was also included in ISPAD guidelines, which adds that the application of this test in CD screening may not be practical nor cost-effective ( 2 ). On the other hand, the NPV of this test in the present study was 100%, a result corroborated by previous studies ( 44 , 45 ). We conclude, therefore, that HLA typing may play a relevant role in cases where both HLA DR3-DQ2 and DR4-DQ8 are negative, excluding the possibility that patients will develop CD, as already suggested by ESPGHAN. However, this is still controversial, with no consensus in the literature, and some studies report the existence of cases of CD whose HLA typing was negative ( 4 , 42 , 45 ).

The follow-up time between the diagnosis of T1D and the diagnosis of CD was an average of 3.40 ± 3.32 years, also in agreement with the literature, that states that most cases of CD are identified in the first 5 years after the diagnosis of T1D ( 17 , 18 , 46 ). This result corroborates the importance of performing CD serological screening, especially in the first 5 years after the diagnosis of T1D, and that must be maintained throughout life.

The gender ratio in the CD group was 1: 1. It is known that CD in general population is more prevalent in females, however, its distribution according to gender in patients with DM1 is variable, with different results in the literature ( 12 , 16 , 18 , 42 , 47 , 48 ).

In our cases with a diagnosis of CD, no GI manifestations were recorded, with only non-GI symptoms occurring. These results are also supported by the current literature, which states that most children and adolescents with T1D and with CD do not develop GI symptoms, presenting with moderate symptoms or even asymptomatic ( 49 – 51 ).

As for average age at diagnosis of T1D, patients with CD had a later diagnosis, without statistical significance and in line with a published prospective study that performed serological screening for CD in 274 patients diagnosed with DM1 for 6 years ( 50 ). However, divergent results were obtained in previously published articles, which demonstrated that children with CD had a lower age at diagnosis of T1D than children without CD ( 12 , 16 , 42 , 52 ). One of the articles concluded that the risk of developing CD is 2.8 times higher in children diagnosed with T1D before 4.5 years, compared with children with a later diagnosis ( 52 ).

Regarding the personal and family history of AID, no statistically significant differences were found between the groups, with and without CD, results also corroborated by the literature ( 52 ).

The small number of patients and retrospective design are the main limitations of this study. It's retrospective design is associated with the impossibility of ensuring a thorough data collection and a smaller data omission in the statistical analysis of some variables. The marked imbalance in the constitution of the two groups that were compared in relation to the variables under study was also a condition for the study. Furthermore, the fact that the mean follow-up time in this population was less than 5 years, prevented the achievement of screening results in all periods recommended for most cases.

In conclusion, this study proved the higher prevalence of CD in a pediatric sample with T1D when compared to general population, with a prevalence 6 times greater than in general pediatric population of the same country ( 15 ). It demonstrated that CD develops, on average, in the first 5 years after the diagnosis of T1D and presents itself predominantly with non-GI symptoms. We also found that serological screening for antibodies associated with CD is an excellent screening test and that HLA typing, although not the most suitable test to be used as first line in screening for CD, can be a useful tool to exclude the possibility of T1D patients developing CD. Thus, it justified the importance of applying CD screening in patients with T1D, so that early detection and intervention is possible, and to guarantee the best metabolic control and the prevention of short- and long-term complications for both diseases.

In order that this screening can be optimized in the future, it is crucial to develop new studies that bring together a consensus regarding the role of HLA typing and that identify factors that are associated with a greater predisposition of patients with DM1 to develop concomitantly CD.
